# Human resources for health during COVID-19: a qualitative analysis of strategies and challenges in Iran

**DOI:** 10.1186/s12913-025-13111-y

**Published:** 2025-07-19

**Authors:** Aref Lotfiyan, Seyed Saeed Tabatabaee, Javad Moghri

**Affiliations:** 1 Student Research Committee, Mashhad University of Medical Sciences, Mashhad, Iran; 2 Social Determinants of Health Research Center, Mashhad University of Medical Sciences, Mashhad, Iran; 3 Department of Management Sciences and Health Economics, School of Health, Mashhad University of Medical Sciences, Mashhad, Iran

**Keywords:** COVID-19, Health personnel, Iran, Health policy, Qualitative research, Health workforce, Pandemics

## Abstract

**Background:**

The COVID-19 pandemic has imposed unprecedented challenges on healthcare systems worldwide, significantly impacting human resources for health (HRH). This study aims to explore and analyze the policies related to HRH in Iran during the pandemic from the perspective of health experts.

**Methods:**

A qualitative study employing framework analysis was conducted. Data were collected through semi-structured, face-to-face interviews with 18 health experts from Mashhad, Iran, selected via purposive sampling. Interviews were analyzed using thematic analysis guided by the Walt and Gilson policy triangle framework, focusing on policy context, content, process, and actors.

**Results:**

Five main themes emerged: shortage of human resources, motivation of human resources, supportive facilities, financial support, and workforce valuation. Severe staffing shortages, exacerbated by high infection rates among healthcare workers, led to prolonged shifts and compromised care quality. Strategies to address these issues included staff redeployment, volunteer recruitment, and improvement of digital health services. However, insufficient financial incentives and unmet promises of job security diminished motivation, while a lack of supportive facilities and psychological assistance heightened staff burnout.

**Conclusion:**

The study reveals severe challenges for Iran’s healthcare workforce during COVID-19, such as staff shortages, low motivation, and insufficient support, impacting healthcare delivery and worker well-being. It calls for robust policies, including strategic recruitment, enhanced training, and motivational support. Engaging frontline staff is crucial to building a resilient health system, ensuring preparedness for future crises.

**Supplementary Information:**

The online version contains supplementary material available at 10.1186/s12913-025-13111-y.

## Introduction

The COVID-19 pandemic, declared a global health emergency by the World Health Organization (WHO) on March 11, 2020, has presented unprecedented challenges to health systems around the globe. Iran, one of the countries most severely impacted by the outbreak, reported its first cases in Qom city on February 19, 2020 [1]. By May 12, 2025, Iran had reported 704,753,890 confirmed cases, 675,619,811 recoveries, and 7,010,681 deaths [2].

The COVID-19 pandemic has presented unprecedented challenges to health systems worldwide, with human resources for health (HRH) at the forefront. In Iran, the pandemic has not only highlighted but also exacerbated pre-existing HRH issues, necessitating the development of innovative strategies to ensure the effective delivery of healthcare services [3]. This study aims to explore the HRH challenges and strategies in Iran’s response to the COVID-19 pandemic through qualitative structured interviews with health experts.

Before the COVID-19 pandemic, Iran’s health sector had implemented various strategies to achieve universal health coverage (UHC), such as developing new academic disciplines, balancing university admissions based on workforce needs, and enrolling students from deprived areas [4]. However, these strategies were not without challenges. A qualitative study conducted in 2019 identified numerous issues in HRH management, including a lack of a national comprehensive policy, inefficient scientific communities, poor data collection methods, and inadequate planning and management of HRH [5]. Additionally, there were problems with job satisfaction, low salaries, and unclear employment policies, which affected the morale and retention of healthcare workers [5].

The COVID-19 pandemic significantly exacerbated these pre-existing challenges. The rapid spread of the virus led to a surge in hospital admissions, putting immense pressure on the already strained HRH [6]. Healthcare workers experienced burnout due to long working hours and increased patient loads, with many facing psychological pressures from the fear of infection and transmission to their families [7]. By May 2020, more than 10,000 medical and healthcare staff were infected, and over 100 had deceased, further depleting the workforce and adding to the emotional burden [8]. Particularly affected were intensive care unit (ICU) nurses, who faced significant occupational challenges including payment system issues, human resource management problems, and psychological distress [9].

In response to these challenges, the Iranian Ministry of Health and Medical Education (MoHME) implemented a range of strategies. These included staff transfer from non-clinical to clinical roles, volunteer recruitment, continuous education on infection control, flexible scheduling to manage staff fatigue, and the use of digital health services for remote care. Financial benefits and mental health support were also provided to maintain staff well-being [10].

Despite these efforts, there is a notable lack of comprehensive qualitative analysis from the perspective of health experts regarding the HRH challenges and strategies during the COVID-19 pandemic in Iran. Existing literature includes studies on specific aspects, such as workload and mental health [11], and general overviews of Iran’s health system response [12] However, a deeper understanding of those directly involved is missing, along with a lack of comprehensive qualitative analysis from the perspective of health experts.

The COVID-19 pandemic has underscored the critical role of HRH in maintaining health system resilience, particularly in low- and middle-income countries (LMICs) where resources are often limited. Iran’s experience offers valuable insights into managing HRH during a crisis, given its unique geopolitical and cultural context. International sanctions have constrained Iran’s access to medical supplies and equipment, thereby exacerbating the challenges faced by its healthcare workforce [13].

Pilgrimages and mass gatherings have been identified as potential superspreading events, as seen globally during the pandemic’s early waves. For example, religious gatherings in Malaysia and Saudi Arabia were linked to spikes in cases, necessitating strict mitigation measures such as scaling down events and implementing health protocols to prevent outbreaks [14].

In Iran, historical parallels show that religious mobility during pandemics, such as cholera, exacerbated disease spread due to inadequate public health infrastructure and quarantine measures. This highlights the challenges posed by pilgrimage sites in managing infectious diseases [15].

On the positive side, religious leaders and organizations have played a crucial role in disseminating culturally sensitive health information, encouraging preventive measures, and fostering community engagement in combating the pandemic [16].

By examining Iran’s HRH strategies and challenges, this study contributes to the global discourse on effective workforce management in crisis situations, aligning with the World Health Organization’s Global Strategy on Human Resources for Health: Workforce 2030 [17]and offers lessons that can be applied to other LMICs facing similar constraints. This study is part of a larger research initiative examining Iran’s health system response to COVID-19, with a specific focus on qualitative perspectives from health experts, complementing quantitative analyses and regional case studies.

This study aims to address this gap by conducting semi-structured qualitative interviews with health experts to explore the key HRH challenges and strategies in Iran’s response to the COVID-19 pandemic. The findings will not only contribute to the academic literature but also inform practical policy-making and preparedness efforts for future health crises.

## Methods

### Study purpose and research questions

This qualitative study aimed to explore HRH challenges and strategies in Iran’s response to the COVID-19 pandemic from the perspective of health experts. The research questions were:

- What were the primary HRH challenges faced during the COVID-19 pandemic in Iran?
- What strategies were employed to mitigate these challenges?
- How effective were these strategies in addressing the HRH challenges?

### Study design and context

Conducted within a constructivist/interpretivist paradigm, this study employed framework analysis to investigate HRH issues. It was part of a broader research initiative examining Iran’s health system response to COVID-19. The study took place in Mashhad, Iran, a city with over 3 million residents and approximately 29 million annual pilgrims due to the eighth Shiite Imam’s shrine. Mashhad’s unique socio-economic and cultural context, including its role as a pilgrimage hub, significantly influenced healthcare delivery and workforce management during the pandemic.

### Participants and sampling

Participants were key informants from Mashhad University of Medical Sciences, defined as individuals with direct involvement in HRH management during the pandemic, including senior managers (e.g., HR directors, department heads), academic staff with expertise in health policy, and operational personnel such as senior nurses. A purposive sampling strategy with maximum variation was implemented to capture a diverse array of perspectives and experiences. Interviews continued until data saturation was reached, defined as the point where no new themes emerged, resulting in a comprehensive and representative dataset. Each interview lasted approximately 32 min on average.

### Data Collection

Data were collected via semi-structured, face-to-face interviews. While face-to-face interviews were favored for building rapport and ensuring nuanced communication, all interviews were conducted in accordance with local health guidelines, including the use of masks, physical distancing, and in well-ventilated spaces.

An interview guide was carefully developed through an extensive review of relevant HRH policy documents and aligned with the Walt and Gilson policy triangle framework (content, context, process, actors). It featured open-ended questions such as, “Can you describe the primary HRH challenges faced during the COVID-19 pandemic in Iran?” and “What strategies were employed to mitigate these challenges?” With participants’ informed consent, all interviews were audio-recorded and transcribed verbatim to ensure the accuracy and depth of the collected data.

### Data analysis

The transcribed interviews were imported into MAXQDA software (version 20) for analysis. A framework analysis approach was utilized, involving the systematic coding of data according to the policy triangle framework’s components. Codes were iteratively grouped and refined into higher-level themes and subthemes, ensuring that identified patterns were robustly supported by the data. Discrepancies in coding were resolved through team discussions, enhancing the reliability and richness of the analysis.

### Ethical considerations and trustworthiness

The study adhered to the Consolidated Criteria for Reporting Qualitative Research (COREQ) guidelines to ensure methodological rigor and transparency [18]. Trustworthiness was upheld by addressing Lincoln and Guba’s criteria:

- *Credibility* was strengthened through meticulous participant selection and triangulation of data sources, and member checking where possible.
- *Dependability* was ensured by maintaining detailed documentation of all research processes.
- *Transferability* was supported by providing thick descriptions of the study context, allowing readers to assess the applicability of findings to other settings.
- *Confirmability* was achieved through a transparent audit trail of coding and analytic decisions and by involving multiple researchers in the analysis.

Ethical approval was obtained from the institutional review board of Mashhad University of Medical Sciences, and informed consent was secured from all participants before their involvement. This robust methodological framework addresses key aspects of the study design, participant sampling, data collection, and analysis, offering valuable insights into HRH challenges and strategies during Iran’s COVID-19 response while meeting established qualitative research standards.

## Results

This study, part of a broader research initiative assessing COVID-19 policies in Iran, focuses on human resource challenges and strategies during the pandemic. Data were obtained from in-depth semi-structured interviews with 18 key informants from Mashhad University of Medical Sciences, including senior managers (e.g., HR directors, department heads), academic staff with health policy expertise, and operational personnel such as senior nurses (Table 1 demonstrates characteristics of the interviewees).

**Table 1:** Characteristics of the study participants

Row	Organizational Responsibility	Education Level	Gender
1	Former director of university health affairs	Ph.D. in Health Promotion	Male
2	Former vice chancellor of medical sciences university	Ph.D. in Health promotion	Male
3	Epidemiologist and faculty member medical sciences university	Ph.D. in Epidemiology	Male
4	Director of university treatment affairs	M.Sc. in Nursing	Male
5	Hospital director & Director of University Laboratory Affairs	M.Sc. in Laboratory Sciences	Male
6	Head of the research and development office of the University of Medical Sciences	Ph.D. in Health Economics	Male
7	Human resources manager of a university hospital	Ph.D. in Health Services Management	Male
8	Director of statistics and IT of the university	M.Sc. in IT	Male
9	Physician	Ph.D. in Clinical Internal Medicine	Male
10	Hospital manager	General Practitioner (M.D.)	Male
11	Head nurse	B.Sc. in Nursing	Female
12	Nurse	B.Sc. in Nursing	Female
13	Nurse	B.Sc. in Nursing	Male
14	Nurse	B.Sc. in Nursing	Male
15	Nurse	B.Sc. in Nursing	Female
16	Nurse	B.Sc. in Nursing	Female
17	Nurse	B.Sc. in Nursing	Female
18	Director of treatment supervision & accreditation of the university	Ph.D. in Medicine	Male

Using framework analysis aligned with the Walt and Gilson policy triangle framework (content, context, process, actors), we identified “Human Resources” as the major theme, with three sub-themes: “Shortage of Human Resources,” “Governmental Human Resources Motivation,” and “Attention and Appreciation for Personnel.” (Table 2).

**Table 2:** Qualitative framework analysis results

Theme	Sub-Theme	Items
Human Resources	Shortage of Human Resources	Shortage of Personnel and Efforts to Compensate for the Shortage
		Impact of Staff Shortages
		Lack of Trained Personnel
	Governmental Human Resources Motivation	Supportive Facilities
		Financial Support
	Attention and Appreciation for Personnel	Positive and Negative Community Actions and Reactions
		Criticism of Insufficient Attention to Healthcare Workers
		Psychological Support in Hospitals

## Shortage of human resources

One of the main issues faced by the health system during the COVID-19 pandemic was the shortage of human resources. Although a shortage of human resources has always been a challenge in some specific sectors of Iran’s health system, this issue became more prominent and problematic during the COVID-19 pandemic due to a sharp increase in the demand for health services. Despite universities’ efforts to supplement health system staff from various departments, these measures were insufficient to bridge the gap and address the critical issue of human resource shortages during the fight against COVID-19.

### Shortage of personnel and efforts to compensate for the shortage

The shortage of human resources presented a serious challenge for managers and health personnel. One interviewee said: “We were so short-staffed that most of our staff were working 12-hour and 18-hour shifts.” (P10).

Another interviewee highlighted the persistent problem of human resource shortages and its exacerbation during crises:

“We always have a shortage of human resources, a shortage of nurses… During crises, the stress and the increase in critically ill patients make us need more nurses and human resources… which unfortunately is never fulfilled.” (P12).

One factor that exacerbated the shortage of human resources was the spread of COVID-19 among healthcare staff. During peaks, a significant portion of the frontline medical staff became infected. One specialist commented on the infection rates at a major teaching hospital during a peak in the pandemic: “…we had 283 staff infected in one day during the peak… They were either at home in quarantine or… in recovery but not fully well… we couldn’t afford to let our staff take more than two weeks of sick leave.” (P10).

Another interviewee described the situation at another hospital: “At the start of the peak, you might have had 400 nurses… but by the end, you wouldn’t even have 100 left… The severity of the disease increased due to high exposure, leading to complications like internal bleeding.” (P5).

An interviewee spoke about the university’s efforts to inject human resources into the health system: “The university tried to address the shortage by recruiting staff… through volunteer forces. Although these weren’t highly specialized personnel, they were helpful during the initial peaks… especially in primary patient care.” (P4).

### Impact of staff shortages

The shortage of human resources also made certain supportive policies, like granting leave to medical staff, ineffective due to insufficient personnel. One interviewee said: “…supportive policies for staff weakened was the shortage of human resources… if our personnel wanted to take their leave, we would face a shortage… increasing the workload of the remaining staff and compromising their safety and security.” (P7).

Nurses were particularly affected by the shortage of staff during the pandemic, experiencing significant physical, mental, and emotional stress. One interviewee said: “The psychological harm and even physical injuries that staff suffered were issues we had to endure while still having to care for patients.” (P11).

### Lack of trained personnel

According to specialists, many deaths from the disease could have been prevented with adequately trained personnel and sufficient equipment. One interviewee said: “We lost so many patients unnecessarily due to a lack of equipment, facilities, drugs, and staff, as well as inexperienced staff.” (P14).

Another interviewee commented on the lack of experience among the personnel sent to help: “They would send us nurses and assistants, but sometimes these people had never worked in ICUs. ICU is a place that requires only trained and specialized personnel.” (P16).

## Governmental human resources motivation

Undoubtedly, one of the most crucial factors for high human resource efficiency is sufficient motivation to provide services. Various support mechanisms and factors can either enhance or diminish the motivation of healthcare workers on the frontline against COVID-19. In this study, we examine these factors under two subheadings: “Financial Support,” and “Supportive Facilities,”.

The specific conditions of the pandemic and the widespread fear and anxiety significantly impacted the motivation and capability of human resources to provide services, making incentive packages essential. One interviewee described the physical and psychological pressures due to the pandemic: “The biggest problems during the COVID-19 pandemic were the illnesses of our colleagues, both mentally and physically. In addition to supporting patients in the hospital, they had to care for sick family members at home, adding to their stress and psychological pressure.” (P11).

Despite considering support packages to increase staff motivation, effective measures were not observed. One interviewee said: “The university was under pressure and facing many shortages. To encourage staff to cope with the situation, they made promises to motivate them, like reducing shifts, allowing leave to be saved, promoting job ranks, and offering job security for contract staff. But none of these promises were fulfilled.” (P17).

Specialists in this field generally viewed the health system as lacking effective policies to increase human resource motivation. One specialist said: “…we didn’t see supportive policies… A good incentive package for the staff would have been beneficial, but unfortunately, it wasn’t provided.” (P7).

### Supportive facilities

Some supportive policies, such as granting incentive leave, were implemented. However, these leaves were not mandatory, and some staff chose to save them for the following year, which defeated the purpose of allowing staff to stay away from COVID-19 environments. One specialist commented: “There were supportive policies like incentive leave. These leaves were good but not mandatory, so people would save them for the next year.” (P7).

Another policy was to grant 14 days of sick leave for staff infected with COVID-19, with social security covering only a third of their salary, and with delays of several months. To address this, a policy was introduced to alternate sick leave with regular leave, but it was complex and insufficient. The specialist continued: “…they had to take 14 days of sick leave, but social security only covered a third of their salary, with months of delay… we introduced a policy where staff would alternate three days of sick leave with three days of regular leave, but this policy was not very effective.” (P7).

The poor implementation of these policies and the unfulfilled promises negatively impacted staff motivation. One interviewee shared their personal experience: “They gave us incentive leaves, but after a year, they removed them from the system, deducting them from my regular leaves, putting me in the negative. They said they made a mistake in granting those leaves.” (P13).

A motivational package for converting contract staff to permanent employees was promised but never implemented. One interviewee said: “Many colleagues were hired on contracts or as temporary staff during the crisis with promises of permanent employment, but none of these promises were kept.” (P12).

Undoubtedly, the unfulfilled promises and empty slogans from officials significantly damaged the motivation of the frontline fighters against the disease. One interviewee said: “As a nurse, I felt obligated to work with these patients, and I expected society to motivate me, not just with empty slogans. At least half or a third of the promises should have been fulfilled, but they weren’t.” (P14).

### Financial support

One of the motivational policies announced by the ministry was the “COVID-19 allowance” to financially motivate staff dealing with COVID-19. Financial incentives, along with welfare and psychological incentives, are undoubtedly important. One specialist said: “The next question was how to motivate them. Welfare discussions were raised, but we must accept that money is always significant. No matter what, financial incentives must be considered.” (P6).

However, according to specialists and frontline staff, the financial incentives offered by universities were ineffective and sometimes perceived as insulting. One specialist said: “…some financial support policies were extremely insulting… a nurse working 250–300 hours in the COVID-19 ward got paid 600–700 thousand tomans (10–12 USD)… these amounts do not motivate personnel… some said it would have been better if we received nothing; we worked for the sake of God.” (P7).

“Our overtime pay wasn’t correct. After a year, overtime became like exploitation; you had to work for free for the system. This lowers motivation and service quality.” (P13).

## Attention and appreciation for personnel

Emotional attention and appreciation from society and managers for the personnel of an organization are significant in increasing employee satisfaction and participation and improving organizational relationships. This leads to improved organizational performance and increased motivation for employees to work in the organization. This sub-theme has comprised of four different items: positive community actions, negative reactions due to fear, criticism of insufficient attention to healthcare workers, and psychological support in hospitals.

### Positive and negative community actions and reactions

Some community members took very positive and encouraging actions to motivate the staff. One interviewee said: “Benefactors and people tried to uplift us with small gestures, like bringing snacks for the staff, or representatives from the shrine would come and encourage us. This made us feel that people were aware of us and appreciated our efforts.” (P12).

However, due to widespread fear and panic caused by the disease, some members of the community unintentionally displayed unkind behavior towards healthcare personnel. An interviewee said: “When we got in a car, for example, and they realized we were nurses, they would distance themselves from us. Relatives also stayed away from us, even though we were being careful ourselves.” (P14).

### Criticism of insufficient attention to healthcare workers

Healthcare workers also criticized the lack of sufficient attention to human resources in the health sector. One interviewee said: “In the context of disease detection, screening, and providing primary level services, unfortunately, the health sector never paid special attention to its human resources.” (P1).

### Psychological support in hospitals

Some hospitals took proactive steps to provide psychological support and emotional care for their staff. For instance, they utilized psychologists and psychiatrists to mitigate the psychological harm experienced by personnel. One interviewee described the psychological support at a hospital: “…our nurses had mental health issues; they were psychologically distressed… we brought in psychologists and psychiatrists, held educational programs and sometimes psychotherapy sessions… we referred our staff to colleague psychologists.” (P5).

Another interviewee talked about setting up a psychological clinic at another hospital: “We set up a psychological clinic to provide mental support for some families and some patients.” (P10).

Table 3 shows the thematic map of HRH findings and the relation of themes and subthemes with the components of policy triangle framework, along with key insights for each subthemes.

**Table 3:** Thematic map of HRH findings

Theme	Subtheme	Framework Component	Key Insight
Shortage of Human Resources	Personnel Shortages	Context, Process	Chronic shortages are worsened by infections (P10).
	Impact of Shortages	Process	Hindered leave policies, increased stress (P7, P11).
	Lack of Trained Personnel	Process	Inexperienced staff led to suboptimal care (P14, P16).
Governmental Motivations	Supportive Facilities	Content, Actors	Unfulfilled promises reduced morale (P7, P12).
	Financial Support	Content	Inadequate incentives demotivated staff (P7, P13).
Attention and Appreciation	Community Actions	Context	Mixed reactions: appreciation and fear (P12, P14).
	Insufficient Attention	Actors	Lack of sector attention to HRH (P1).
	Psychological Support	Process	Mental health support provided (P5).

## Discussion

The COVID-19 pandemic has starkly highlighted the critical challenges facing human resources for health (HRH) in Iran, exacerbating pre-existing issues and creating new ones. This discussion delves deeper into these challenges, drawing on specific data, global comparisons, and structured policy recommendations to provide a comprehensive analysis of the implications for Iran’s health system and beyond.

### Summary of key findings

The qualitative analysis revealed three primary HRH challenges in Iran during the COVID-19 pandemic: severe shortages of healthcare personnel, inadequate governmental strategies to motivate the workforce, and varied levels of attention and appreciation from the community and health authorities. These challenges were exacerbated by high infection rates among healthcare workers, leading to further depletion of an already strained workforce. Efforts to address shortages, such as recruiting volunteers and transferring staff, were insufficient. Motivational policies, including financial incentives and promises of job security, were often unfulfilled or perceived as inadequate, negatively impacting staff morale. Community responses ranged from supportive gestures to fear-driven avoidance, reflecting complex societal dynamics.

### Severity of HRH shortages and their impact

The COVID-19 pandemic severely strained Iran’s healthcare workforce, with over 10,000 infections and 100 deaths among healthcare workers by May 2020 [8]. This alarming statistic highlights the critical staff shortages that plagued the system during the crisis. Pre-existing systemic issues, such as inefficient distribution of human resources and compensation disparities [19]. During the pandemic, these shortages led to extended work shifts, often 12 to 18 h, as reported by interviewees, and compromised the quality of care, particularly in intensive care units (ICUs) where untrained personnel were sometimes deployed.

The physical, mental, and emotional toll on healthcare workers, especially nurses, was significant, with increased burnout and stress reported [20].

The pandemic worsened Iran’s healthcare system’s existing problems, including financial constraints that caused unequal pay, staffing issues, and unbalanced workloads. A study pointed out poor coordination, lack of clear health protocols, and weak performance evaluations as key reasons for employee dissatisfaction and high turnover [21].

Support measures during the crisis failed to boost morale, with many seen as inadequate or even offensive. Financial rewards were insufficient, and unkept promises of job security further eroded trust in health officials. To maintain morale and performance in crises, meaningful actions like fair pay, manageable workloads, and genuine recognition are crucial [22].

These challenges emphasize the urgent need for robust human resources for health (HRH) policies to address both immediate crises and long-standing systemic vulnerabilities.

### Global comparisons and lessons learned

Iran’s experience with HRH challenges during the pandemic offers valuable lessons when compared to other countries. South Korea’s tech-driven approach, including digital contact tracing and telemedicine, effectively reduced the burden on healthcare workers by minimizing unnecessary exposures and optimizing resource allocation [23]. In contrast, Iran’s response relied heavily on in-person care despite resource constraints, which may have contributed to higher infection rates among staff. The United States also faced significant HRH shortages, particularly in hotspot areas, and implemented strategies such as deploying retired healthcare workers and utilizing federal support to bolster the workforce [24]. These measures provided additional manpower that Iran could consider adapting, such as creating a reserve pool of healthcare professionals.

Similarly, in Latin American and Caribbean countries, the COVID-19 pandemic led to significant disruptions in health services due to insufficient staff availability. A study analyzing policy responses in these regions highlighted the importance of flexible working arrangements, mental health support, and training for infection control to protect and motivate the healthcare workforce [25]. These strategies align with some of the recommendations from our study participants, emphasizing their global relevance.

Furthermore, the World Health Organization has issued comprehensive guidelines for protecting healthcare workers during the COVID-19 pandemic, focusing on infection prevention and control, adequate personal protective equipment, and mental health support [26]. Implementing such guidelines is crucial for safeguarding the health workforce, as evidenced by the high infection rates in Iran where these measures may have been insufficient.

These comparisons underscore the importance of context-specific strategies while highlighting the universal need for robust HRH management during crises. Iran’s challenges were exacerbated by long-standing systemic issues, such as poor working conditions and inadequate training, which were not solely pandemic-related but deeply rooted in the health system’s structure [5].

## Conclusion

The COVID-19 pandemic exposed critical human resources for health (HRH) vulnerabilities in Iran, including acute staff shortages, diminished motivation, and mixed community responses. With over 10,000 healthcare workers infected and 100 fatalities by May 2020 [27]. the crisis highlighted the urgent need for robust HRH systems. This study, employing the Walt and Gilson framework, systematically analyzed these challenges and proposed actionable solutions, contributing valuable insights for Iran and similar resource-limited settings.

While the focus on Mashhad provides in-depth understanding of a unique context, it may limit the generalizability of findings to other regions. Nevertheless, the identified challenges and strategies offer lessons applicable to other low- and middle-income countries (LMICs) grappling with similar HRH issues during health emergencies.

To build a resilient HRH system, Iran should consider innovative approaches such as leveraging digital health technologies for training and service delivery, establishing a flexible workforce model that can adapt to crisis demands, and fostering international collaborations to share best practices and resources. Additionally, investing in mental health support and fair compensation for healthcare workers is crucial to sustain motivation and retention.

By integrating these strategies and learning from both domestic and global experiences, policymakers can enhance Iran’s health system preparedness for future crises, ultimately improving healthcare delivery and population health outcomes.

### Recommendations

To address these challenges, policymakers must adopt a structured approach, guided by the Walt and Gilson policy triangle framework, which considers policy content, context, process, and actors.

- Content: Policies should focus on increasing the healthcare workforce through recruitment and retention programs.
- Context: Given Iran’s economic constraints, policies must prioritize efficient resource allocation to support HRH initiatives.
- Process: Proactive policy implementation is essential, including the development of scalable workforce strategies, such as establishing a reserve healthcare workforce that can be rapidly deployed during crises.
- Actors: Improved coordination among government agencies, healthcare institutions, and international organizations is critical to ensure a cohesive and effective response.

In addition to these framework-specific recommendations, other key actions include:

- Fair Pay and Recognition: Implementing fair financial incentives for healthcare workers.
- Mental Health Support: Providing consistent psychological support, as outlined by the WHO’s Mental Health and Psychosocial Support for COVID-19 is vital for maintaining staff well-being.
- Community Engagement: Launching public awareness campaigns, akin to the UK’s “Clap for Carers”, can foster community support and reduce stigma against healthcare workers.
- Leverage Technology: Adopt digital health solutions, such as telemedicine and digital training platforms, to reduce the burden on healthcare workers and enhance service delivery efficiency.

### Future preparedness

Looking ahead, Iran must invest in building a resilient HRH system by addressing both immediate and systemic challenges. This includes not only expanding the healthcare workforce but also ensuring that staff are adequately trained, supported, and valued. By learning from both domestic experiences and global best practices, policymakers can develop a health system that is better prepared for future crises, ultimately improving both healthcare delivery and population health outcomes.

## Supplementary Information

Supplementary Material 1.

## Data Availability

The datasets used and/or analyzed during the current study are available from the corresponding author on reasonable request.

## Abbreviations

HRH: Human Resources for Health
WHO: World Health Organization
UHC: Universal Health Coverage
ICU: Intensive Care Unit
MoHME: Ministry of Health and Medical Education
LMICs: Low and Middle Income Countries
