# Supplementary material for: Human resources for health during COVID-19: a qualitative analysis of strategies and challenges in Iran
Source: BMC Health Serv Res. 2025 Jul 19;25:961. doi: 10.1186/s12913-025-13111-y (PMC12275368; doi:10.1186/s12913-025-13111-y)
Supplement: Supplementary file 1 — Supplementary Material 1. [file 12913_2025_13111_MOESM1_ESM.pdf]

Interview Guide: HRH Strategies and Challenges During COVID-19 in Iran

Purpose: To explore HRH challenges, mitigation strategies, and policy impacts during the pandemic.

1. Introduction & Consent

- Brief introduction to the study.
- Confirm consent for audio recording and anonymity.

2. Demographic Information

- Role, years of experience, and facility affiliation during COVID-19.

3. HRH Challenges (Content & Context)

1. **Shortages**:
   - How did COVID-19 exacerbate HRH shortages in your facility?
   - What strategies were used to address shortages (e.g., redeployment, volunteers)? Were they effective?
   - How did staff infections impact workforce availability?
2. **Skill Gaps**:
   - Were there challenges with untrained personnel in critical areas (e.g., ICUs)? How did this affect care?

4. Mitigation Strategies (Process & Actors)

3. **Motivation & Support**:
   - What financial (e.g., hazard pay) or non-financial (e.g., leave, psychological support) incentives were provided?
   - Were promises (e.g., job security, promotions) fulfilled? If not, how did this affect morale?
4. **Policy Implementation**:
   - How effective were policies like sick leave, overtime pay, or staff recruitment? What barriers existed?

5. Community & Organizational Dynamics (Actors)

5. **Public Perception**:
   - Did you experience community support or stigma? How did this impact your work?
6. **Managerial Response**:
   - How did hospital leadership address staff well-being (e.g., mental health support)?

6. Closing Reflections

7. **Lessons Learned**:
   - What key policy changes would improve HRH resilience in future crises?

Format: Semi-structured, open-ended.
Probing: Follow up with *"Can you elaborate?"* or *"How did this affect staff/patients?"*
